# Multidrug Resistance in Cancer Cells: Focus on a Possible Strategy Plan to Address Colon Carcinoma Cells

**DOI:** 10.3390/life12060811

**Published:** 2022-05-30

**Authors:** Chenmala Karthika, Raman Sureshkumar, Mehrukh Zehravi, Rokeya Akter, Faraat Ali, Sarker Ramproshad, Banani Mondal, Milton Kumar Kundu, Abhijit Dey, Md. Habibur Rahman, Angela Antonescu, Simona Cavalu

**Affiliations:** 1 Department of Pharmaceutics, JSS College of Pharmacy, JSS Academy of Higher Education & Research, Ooty 643001, India; karthika1994haridas@gmail.com; 2 Department of Clinical Pharmacy Girls Section, Prince Sattam Bin Abdul Aziz University, Alkharj 11942, Saudi Arabia; mahrukh.zehravi@hotmail.com; 3 Department of Global Medical Science, Wonju College of Medicine, Yonsei University, 24, Wonju 26426, Korea; rokeyahabib94@gmail.com; 4 Department of Licensing and Enforcement, Laboratory Services, Botswana Medicines Regulatory Authority (BoMRA), Gaborone 999106, Botswana; frhtl6@gmail.com; 5 Department of Pharmacy, Ranada Prasad Shaha University, Narayanganj 1400, Bangladesh; ramproshad131135@gmail.com (S.R.); banani091110@gmail.com (B.M.); 6 Pharmacy Discipline, Khulna University, Khulna 9208, Bangladesh; miltonkundu@gmail.com; 7 Department of Life Sciences, Presidency University, Kolkata 700073, India; abhijit.dbs@presiuniv.ac.in; 8 Faculty of Medicine and Pharmacy, University of Oradea, Pta 1 Decembrie 10, 410087 Oradea, Romania; antonescu.angela@yahoo.com

**Keywords:** cancer, P-glycoprotein, curcumin, lipids, colon cancer, multidrug resistance, 5-fluorouracil

## Abstract

Even though various treatment methods are available for cancer, the death curve is not reducing. The diagnosis of cancer at the fourth stage and drug resistance are the leading reasons for treatment failure and lower survival rates. In this review article, we summarize the possible pitfalls during cancer treatment in general, which mainly include multidrug resistance, and propose a hypothesis for colorectal cancer specifically. We also evaluate multidrug resistance in cancer in general and colorectal cancer in particular and hypothesize a concept based on combination therapy with 5-fluorouracil, curcumin, and lipids for the possible management of colorectal cancer. In addition, a hypothetical approach, combining a synthetic agent and a natural chemotherapeutic agent, to treating colorectal cancer is also discussed. This hypothesis could improve the management of colorectal cancer.

## 1. Introduction

Cancer causes cells to divide uncontrollably. This can result in tumors, damage to the immune system, and other impairment that can be fatal. Although various cancer treatment methods are available, the death curve is not reducing [1]. Scientists are researching an alternative delivery system for drug administration due to the pain related to conventional delivery approaches. Various studies estimated that the majority of patients (89%) prefer oral medication compared to intravenous or injection delivery [2]. Better patient compliance, reduced costs, increased tolerability, greater safety, and, possibly increased efficacy, are the main reason for the increase in focus on the oral delivery of anti-cancer drugs. However, the oral bioavailability of anticancer drugs is inadequate because of idiosyncratic physiochemical properties and biological barriers, such as pre-systemic metabolism and gastrointestinal instability [3]. Cancer, also known as malignancy, is a condition characterized by abnormal cell growth. The most common types of cancer are breast cancer, skin cancer, lung cancer, prostate cancer, and colon cancer, in addition to over 100 others. The symptoms of cancer are determined by the cancer type and origin. Colorectal cancer is any development, lump, or tumor that develops in the colorectal area [4]. It is also identified as rectal cancer, colon cancer, or bowel cancer. Colorectal cancer is the world’s third most common cancer after lung cancer, according to the Centre for Development Studies (CDS) and the World Health Organization (WHO), with approximately 1.4 million new cases registered in 2012. This figure is predicted to rise to 24 million by 2035 [5]. Colorectal cancer affects one out of every twenty people in the United States, according to the American Cancer Society. Men and women are equally affected by colorectal cancer, but men are more likely to develop it when they are younger. Although the precise causes of colorectal cancer are indefinite, experts believe that diet plays an important role [6]. The invasion of cancer cells into different layers determines the stages of colon cancer. Metastasis, or the spread of cancer cells to further parts of the body, is most common in the fourth stage, or the Duke E stage, and is caused by cancer cells spreading through the lymph nodes, which is also the main reason for the disease’s reappearance after treatment [7].

The treatment options for colorectal cancer, which include surgery, chemotherapy, and radiotherapy, are primarily determined by the tumor’s current stage and size, as well as the patient’s overall health. Cancer, in most cases, is recognized at the later stage, when metastasis is already underway. If this occurs, the survival rate is less than 15%. Multidrug resistance by cancer cells is the major issue that is faced during therapy [8].

Below, we present the resistance components of cancer in general and colorectal cancer in particular and postulate a concept that includes combination therapy with 5-fluorouracil, curcumin, and lipids for the potential management of colorectal cancer [9]. In addition, we present a hypothetical approach combining a synthetic drug with a natural chemotherapeutic agent to treat colorectal cancer. In a hypothetical scenario, this may be an idea that could lead to the management of colorectal cancer.

### 1.1. General Approach and Evolution of Multidrug Resistance

For most metastatic cancer cells, chemotherapy with classical or targeted anticancer drugs mostly results in remission, but there is less chance of curing cancer with this approach. Cancer cell populations can be intrinsically resistant, meaning they do not respond to anti-cancer drugs at all; or, even if they are essentially sensitive to treatment, they can be resistant to some drugs over the course of therapy thereby acquiring resistance [10].

There are two leading hypothetical approaches to demonstrating the evolution of multidrug resistance, which usually results in treatment failure [11].

1. Within the primary group of the tumor cells, there are limited cells, perhaps small subgroups, which are already resistant to the treatment methodology. There are indications of this in acute myelogenous leukemia, and it is certainly the case in melanoma. When tumors are treated and remission occurs, the cells that regrow are originally derived from the parent tumor cells. This has been demonstrated using sophisticated molecular techniques [12].
2. There may be resistant insensitive cells in the initial group, but during treatment, an additional mechanism of resistance can become evolved, which may become fixed during treatment, leading to mixed resistance [13].

Although these appear to be complementary approaches, the mechanisms used to deal with these types of resistance are different. In the first case, a method for delivering therapy that kills as many kinds of resistant cell as possible in the first treatment is needed. In the second case, it is not possible to determine which type of resistance will originate. Hence, the therapy should be personalized for each tumor, based on the identification of the mechanism of action shown by the tumor cells of the individual patient [14]. This can make the therapeutic regimen much more difficult [15]. In most cases, this is the major reason for the development of resistance [16]. For researchers, the goal is to understand these mechanisms at the molecular level in order to determine mechanisms that operate in every case. Targeted therapies aimed at specific oncogenes, can be used against tumors that are often found to have mutations in their targets or to bypass the mutations al together [17]. For example, if the target region is a growth-factor receptor, and the drug no longer inhibits the specific targets, an alternative approach should be validated to activate the tumor using a different kind of pathway from the one that is targeted. Furthermore, cells can show altered cellular level pharmacology. This mechanism has been studied in considerable detail and has enabled the development of an understanding of how drugs are handled in the body, based on how cancer cells handle these drugs [18]. This includes mechanisms that can reduce the accumulation of drugs, alter the metabolism of drugs, or increase the efflux of drugs from cells. The third alteration, is the most important in the development of drug resistance changes in the differential pathway and homeostasis responses within tumors [19]. Therefore, a tumor that begins as an epithelial kind can be adverse and further evolve into a mesenchymal tumor, with massive changes in gene expression, resulting in a very different pattern of resistance to anti-cancer drugs. A classic example is the transition of epithelial to mesenchymal cells. This ultimately results in deviations in the local environments of tumor cells, including the development of other cell types, which are not cancerous but can affect responses to chemotherapy, as well as alterations in the substrates where the cells sit [20]. Additional effects include changes in the tissue elasticity and plasticity, alterations in the immune mechanism, and alterations in the mechanism that determines the growth of blood vessels into tumor cells. All of these effects are associated with drug resistance, but most studies carried out on this topic evaluate the changes associated with cellular pharmacology [21].

If the cell-based mechanism of drug resistance is taken into consideration, then there are three general classes of alteration in tumors that can result in the reduced efficacy of chemotherapy. The first, and most obvious, is when drugs simply do not enter cells; nearly 400 different solute carriers are responsible for the movement of normal nutrients [22]. Other agents act within cells, and approximately 10% of them have been demonstrated to affect the uptake of specific drugs into cells; reductions in the amount or changes in the specificity of these transporters can produce drug-resistant cells [23]. If the drugs can enter the cells, some mechanisms affect the cell biology, which has profound effects on drug resistance, such as reduced cell death by pathways, known as apoptosis, altered cell-cycle checkpoints or growth pathways, the altered metabolism of drugs within cells, altered targets, or increased damage repair [24]. Even the compartmentalization of drugs within subcellular compartments prevents them from reaching their targets [25].

The major mechanism of resistance is related to the increased energy-dependent efflux of drugs from cells, which are then involved in the cloning of some of the transporters that are responsible for the efflux of the drugs in the first place [26]. Furthermore, it was discovered that these belong to the family of ATP-dependent transporters, called ABC transporters, of which four are known in humans [27].

Below is an overview on the development of insights into cancer cells and drug resistance:

The 1940s: The first cancer chemotherapy trials begin.

The 1970s: Mammalian cells resistant to specific antitumor agents frequently showed cross-resistance to drugs that were dissimilar in their structures and modes of action.

Multidrug resistance was a major problem in cancer chemotherapy because it elaborated resistance to some of the most frequently used anticancer drugs.

1980s: Multidrug resistance was shown, in most cases, to result from decreased intercellular drug accumulation, apparently as a result of alterations in the plasma membrane [28].

The major reasons for studying resistance transporters and their activities are [29]:

1. They play a major role in e multidrug resistance in tumor cells.
2. They play a significant role in drug pharmacokinetics (uptake, distribution, and excretion).
3. They play a significant role in drug toxicity.
4. They play a key role in development (stem cells, morphogenesis).
5. To acquire knowledge about the biology of all transport systems.

### 1.2. Multidrug Resistance and Its Mechanistic Approaches

Drug-sensitive cells are killed by chemotherapy, but the major drawback of this approach is that cells that are resistant to the drug are left behind [30]. When the tumor starts to grow again, chemotherapy may fail as the cells become resistant. The main obstacle to giving chemotherapy to patients is multidrug resistance (MDR). The overexpression of P-gp is found in MDR, which results in a greater efflux of chemotherapy in cancer cells [31]. A method to inhibit the P-gp was studied to reduce the MDR in cancer patients, but it showed no satisfactory results. Chemotherapy hits the cells that are sensitive to drugs, but it leaves behind a high percentage of drug-resistant cells. As the tumor cells begin to multiply, chemotherapy fails, since the new tumor cells are now resistant to the therapeutic regimen [32]. MDR occurs when in vitro tumor cells are exposed to a cytotoxic agent that has developed cross-resistance to all the compounds that are unrelated structurally and functionally [33]. The increased expression of particular proteins, such as cell membrane transporters, which leads to a greater efflux of cytotoxic drugs from cancer cells, helps to lowering the intracellular concentration, leads to the development of drug resistance in cancer cells [34].

Many mechanisms lead to the development of MDR, such as increases in the drug efflux from the cells by ATP-dependent transporters, decreases in drug uptake by cells, the activation of detoxifying enzymes, and defective apoptotic pathways [35]. MDR’s etiology may be considered multifactorial, but the resistance to cytotoxic drugs discussed above is mostly due to the increased expression of P-gp, which is a 170KD ATP-dependent membrane transporter that exhibits action as a drug efflux pump. P-gp is part of the ATP-binding cassette (ABC) family of transporters, which currently has 48 members that have sequential and structural homology [36]. The energy released during the hydrolysis of ATP is used by the transporters to transport various molecules across the cell membrane. Most of the transporters are overexpressed in human tumors, in addition to their physiological expression [37].

**Figure 1:**
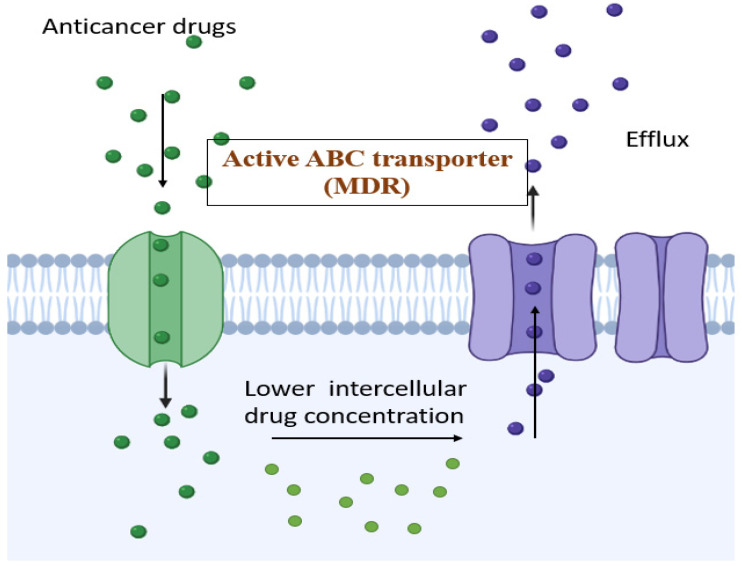
MDR and its mechanism of action—ABC-transporter-mediated drug response.

P-gp was discovered in 1971 by Victor Ling. The P-gp ion is mostly highest in tumors that are derived from tissues that are commonly expressed. P-gp is expressed in the epithelial cells present in the colon, kidney, adrenal pancreas, and liver, which leads to resistance to a few cytotoxic agents before the commencement of chemotherapy in the case of cancerous tissues [38]. However, in other types of tumor, there is decreased expression of P-gp during diagnosis, although it increases when the tissues are exposed to chemotherapeutic agents, which finally leads to the development of MDR in these cancerous cells [39]. Hence, there is an increase in the literature relating to the failure of a few chemotherapeutic agents when they are expressed in P-gp. The mechanism of MDR transporters is given in Figure 1.

The inhibition of the production of P-gp has been studied for more than 20 years as a method of reversing MDR. Several agents that help to inhibit P-gp, such as calcium channel blockers, steroidal agents, protein kinase c inhibitors, immunosuppressive drugs, antibiotics, and surfactants, were found [40]. Hence, now it is known that the chief limitation of the early agents is that they reverse MDR at a very high concentration, which leads to increased toxicity, which cannot be accepted. Cancer MDR is defined as the cross-resistance or insensitivity of cancer cells to the cytostatic or cytotoxic action of numerous anticancer drugs that are structurally or functionally unrelated and have different molecular targets [41].

The factors that usually lead to multidrug resistance are:

(1) The specificity of the individual cells concerning the absorption, metabolism, and delivery of the drugs to the specific targeted tissues. These factors are dependent on the individual’s genetic patterns, which help to regulate the cellular responses, which, in turn, prevent the drug from reaching its threshold levels, which is usually required for pharmacological action to take place [42].
(2) The specificity of the tumor in terms of its origin, vasculature, and tissue function. Resistance to chemotherapy is observed when the tumor is located in the parts of the body where the drug cannot be accessed or the tumor comprises compromised vasculature [43].

## 2. MDR Modulators and the Mechanisms of Inhibitors

MDR modulators are defined as compounds that have the ability to reverse the resistance against anticancer cells. They are also termed MDR inhibitors or chemo-sensitizers. Based on the affinity of the transporter protein, the toxicity of the protein to the normal cells, and the side effects, the modulators are selected. Anticancer drugs are administered along with the inhibitors so that the transporter proteins are kept occupied and the drug is allowed to accumulate in the cancer cells at an effective lethal concentration, as the inhibitors, when considered on a chemical basis, cause intrinsic toxicity or produce undesired effects [44]. Recent research supports the use of natural products as MDR modulators. As the natural compounds used are essential in the human diet, it can be stated that there would be very few side effects [45]. A brief overview of the use of MDR is presented in the section below.

### 2.1. ATP Binding Cassette (ABC)

ATP binding cassette (ABC) transporters are a class of proteins that cause adverse drug reactions by using ATP-dependent efflux pumps. The main cause of MDR is the overexpression of the ABC transporters [46]. Many transport proteins belonging to the ABC family are characterized; they include P-gp, MRP-1, and its homolog, MRP2, which have a higher expression in malignant cells, which leads to the pumping out of the cancer drug from malignant cell, which, in turn, means that lower amounts of the drug are needed to produce therapeutic action. Even if the resistant proteins are from the same ABC subfamily, they are quite different in terms of gene locus amino acid sequence, protein structure, and substrate [47].

Higher levels of P-gp are not the only cause of MDR. Many cell lines that are selected for their resistance do not contain higher levels of P-gp but are usually resistant to a large number of drugs that are obtained from natural sources. In one of these non-P-gp MDR lines, the H69AR small-cell lung carcinoma line, Cole et al. found the amplification and increased expression of a novel gene, MRP (MDR-associated protein) gene [48]. Previous research has proven that MRP is a drug-resistance gene, but the mechanism of action of MRP is unknown. The location of MRP at the subcellular level was not the same as that of the plasma membrane transporter-like P-gp [49]. A protein weighing 190KDa was found in non-P-gp MDR cells, even though the MRP was present in an endoplasmic reticulum other than the plasma membrane [50].

MRP is not usually comparable to the drug transporting P-gp in its actions [51]. As in the case of P-gp, MRP also can exhibit resistance to a range of hydrophobic drugs.

MRP is usually present in the plasma membrane.

MRP can cause decreases in the accumulation of drugs in cells, and this mechanism can be terminated through the permeabilization of the plasma membrane.

The efflux of drug from cells can be increased by MRP.

Even though these are the major differences between MRP and MDR1 P-gp in the drugs that they transport or interact with, the drugs on which these two proteins act are varied, and the inhibitors that modify their activities are also different [52].

Three main multidrug transporters are:

- ABCB1 (P-gp)
- ABCC1 (MRP-1)
- ABCG2 (BCRP, MXR)

### 2.2. BCRP

The breast-cancer-resistant protein is usually present on chromosomes such as P-gp and MRP-1. This BRCP has a wide range of substrate specificities, which are usually different from but overlaps with P-gp or MRP-1 [53]. Due to the functioning and the presence of P-gp and MRP, they are considered the targets for various anticancer efforts. In the fight against cancer, a large number of targets are pursued with the same zeal [54].

BCRP may have a function in shielding the organism from potentially dangerous xenobiotics due to its tissue localization in the placenta, bile canaliculi, colon, small intestine, and brain microvessel endothelium. Human breast cancer (MCF-7), colon carcinoma (S1 and HT29), gastric carcinoma (EPG85-257), fibrosarcoma (EPF86-079), and myeloma sublines were discovered to significantly overexpress BCRP mRNA (8226) [55]. According to these findings, BCRP overexpression was most likely a critical cellular defense mechanism activated in response to mitoxantrone exposure [56].

Miyake et al. (1999) cloned a cDNA from the mitoxantrone-resistant colon cancer cell line S1-M1-80 and named it MXR, since the gene’s expression imparted mitoxantrone resistance. According to the Human Gene Nomenclature Committee, BCRP is the second member of the ABC transporter G (white) subfamily. Brangi et al. (1999) studied the glucuronidation of 4-methylumbelliferone (4-MU), SN-38, mitoxantrone, and epirubicin in the mitoxantrone-resistant colon cell line S1-M1-80 and the doxorubicin-resistant MCF-7/AdrVp 3000 breast cancer cell lines [57]. MCF-7/AdrVp 3000 cells generated higher glucuronides for 4-MU and epirubicin than parental cells. The extent of glucuronidation with each compound was similar in sensitive and resistant colon cancer cell lines that had increased UDP-glucuronosyltransferase activity compared to breast cancer sublines, implying that glucuronidation is not rate-limiting for the resistance mechanism in S1-M1-80 cells [58].

BCRP expression was moderate-to-strong in the placenta, liver canaliculi, colon, small intestine, cardiac muscle, endocrine pancreas, adrenal cortex, thyroid, and parathyroid. The apical membranes of the small intestine and colon epithelium, as well as the bile canalicular membrane, showed strong staining for BCRP [59]. BCRP reactivity was missing or low in bladder cancer, ovarian cancer, and small-cell carcinomas, but was found in colon cancers, esophageal cancers, endometrial cancers, lung malignancies, and melanoma, with cytoplasm and plasma membrane staining identified. If these findings are verified, they will have significant implications for the function of BCRP in clinical oncology [60].

### 2.3. Proteasomes

The 26 s proteasome is a supra-molecular protein assembly that exhibits a major role in degrading the proteins that are involved in the regulation of the cell cycle [61]. Its role is to unfold the protein substrate and stimulate proteolytic activity. This proteasomes is responsible for providing cells with a recycler function in damaged or misfolded proteins [57]. This function has a major role in the regulation of the cell cycle and apoptotic pathways. The p53 tumor-suppressor protein is one of the important biological targets of proteasomes [62]. The functions of this proteasome are to restrict the growth of cells or to induce cell death. As the levels of the P53 suppressor are consistently low due to the ubiquitin proteasomal system, proteasomal inhibitors are required [63]. The pharmacological inhibition of proteasomes leads to the increased accumulation of the P53 protein, which finally leads to apoptosis in cancer cells [64].

## 3. Colorectal Cancer: Understanding the Background

When colorectal cancer is detected in its early stages, surgery is the most commonly used treatment [65]. Surgery does not treat the disease; it only relieves the symptoms. Colectomy is performed to eradicate the cancerous portion of the colon, but it can lead to significant clots, blood loss, and infections. Patients may experience swelling, severe pain, and unpredictable emotional or physical stress responses as a result of the treatment methods [66]. Radiotherapy is an additional treatment option that uses advanced radiation energy to kill cancerous cells. It can cause rectal irritation, painful bowel movements, and blood in the stool in some patients [67].

Chemotherapy is a treatment that kills abnormal cells by using chemicals or drug substances. It is mostly used before and after surgery because it shrinks tumor cells and lowers the likelihood of recurrence. Colorectal cancer medications include 5-fluorourcil (5-FU), irinotecan, capecitabine, oxaliplatin, cetuximab, bevacizumab, panitumumab, regorafenib, and others. Chemotherapy attacks drug-sensitive cells while leaving drug-resistant cells alone; however, when tumor cells re-grow, chemotherapy fails because the cells become resistant to the medication. Another reason for chemotherapy failure is the multidrug resistance produced by cancer cells to a wide range of anticancer drugs [68]. The overexpression of P-glycoprotein prevents chemotherapy drugs from entering cancer cells, posing a barrier to treatment success, eventually leading to multidrug resistance. Most cancer cells have become drug-resistant, necessitating the development of a new strategy to overcome this resistance and ensure the success of the therapy [69].

Flavonoids are the most common polyphenolic compound in the human diet, along with the secondary metabolites originating in both plants and animals [14]. Its anticancer action and natural P-gp inhibitory activity have been described in numerous surveys and studies. Because they have anticancer activity, it is thought that when combined with 5-fluorouracil, they have synergistic effects in the human body. The multi-use of lipids provides several advantages, including improved bioavailability, drug targeting to tumor cells, the lymphatic targeting of the drug, which has benefits during the metastatic stage, and abridged toxicity [70].

### 3.1. Outline and the Hypothetic Concept

We propose that our idea could be used to treat colon cancer. 5-FU, the first-line treatment regimen for colorectal cancer, has caused cancer cells to develop resistance. This is one of the primary reasons for the therapy’s failure. Curcumin, a naturally occurring flavonoid, has been shown in numerous studies to have anti-cancer and MDR inhibition properties [71]. Furthermore, flavonoids are well known for their immunomodulatory, antioxidant, anti-inflammatory, and antimicrobial properties, among others. These findings support our hypothesis that flavonoids can be used to enhance the activity of first-line therapy as an antiproliferative agent [72]. Curcumin and 5-FU together have the potential to recover the potency and efficacy of cancer treatment. A significant amount of research suggests curcumin’s chemosensitizing property, which gives it an advantage in addressing this issue. Because lipid-based drug delivery travels through the lymphatic system, including lipids in this combination [73] can help with cancer cell targeting and even treatment during the metastatic stage [74]. As a result, this novel combination has the potential to overcome the issues related to therapy failure and serve as an alternative throughout the metastatic phase of cancer. Because cancer cells spread through the lymphatic system from the site of formation, targeting the lymphatic system may aid in disease suppression. As a result, the treatment mode’s efficacy can be improved [39]. Furthermore, lipid-based nano systems help to suppress P-gp efflux transporters, which helps to reverse MDR-related issues [75].

### 3.2. Hypothetical Approaches

A novel method for targeting colorectal cancer cells that employs a dual combination of flavonoids and chemotherapeutic drugs is discussed below. The concept stated above can be tested experimentally. The most difficult challenge in colorectal cancer treatment is multidrug resistance (MDR). P-gp is the main reason for MDR in cancer cells [76]. MDR is a situation in which cancer cells efflux drugs, as well as other foreign bodies, resulting in lower absorption of the drug inside the cells and ineffective treatment. We test our concept by looking at colon cancer cells’ multidrug resistance to 5-fluorouracil. The overexpression of P-glycoprotein (P-gp) is the primary reason for MDR in colon cancer cells. We address this issue in our hypotheses by combining 5-fluorouracil (5-FU), a commonly used drug against colon cancer, with a flavonoid (curcumin) in lipid-based drug delivery. Flavonoids have also been shown to be effective as chemosensitizers, which can influence drug efficacy [77]. In addition to the aforementioned issues, the causes of therapy failure include rapid drug catabolism, poor absorption, and short biological half-life, all of which can be avoided by converting to lipid drug conjugates in lipid-based drug delivery systems [78]. P-gp is expressed in the intestine and reduces drug absorption as a substrate; it therefore plays an important role in regulating drug distribution and bioavailability. As a result, the drug’s bioavailability and therapeutic plasma concentration do not occur [79]. However, lowering P-gp expression allows the drug to influence therapeutic plasma concentrations. The substrate is transported to P-gp via a cytoplasmic opening or an entrance in the membrane’s inner leaflet [80]. The cytoplasmic side of the protein binds ATP (adenosine triphosphate). The substrate that must be eliminated from the cell changes because of ATP hydrolysis. The substrate is expelled when phosphate is liberated from the initial ATP molecule [81]. A new ATP molecule attaches to the secondary ATP binding site when an adenosine diphosphate (ADP) is released. If the protein is reactivated by hydrolysis and the release of ADP and a phosphate molecule, the process restarts [82]. P-gp activity is inhibited by curcumin.

Curcumin is a flavonoid that occurs naturally and is widely consumed [71]. Flavonoids are absorbed by intestinal cells after passing through the digestive tract. Several studies have shown that flavonoids can kill cancer cells and prevent their growth. The inhibition of enzymes, DNA, and proteins is specifically targeted. Natural inhibitors have a greater potential impact on safety, efficacy, and cost than chemical or synthetic inhibitors [83]. The synergistic effect of flavonoids can overcome the limitations on most anticancer medications caused by multidrug resistance [84].

Curcumin has also been shown to be effective at preventing P-gp-induced MDR [85]. It inhibits P-gp via the P13K/Akt/NF-kB pathway in mouse MDR leukemia L1210 cells, according to studies. When adriamycin and curcumin were combined, the Western blotting results showed that it could cleave PARP inhibitors and overcome P-gp-induced MDR. Curcumin has previously been shown, in animal studies and cell culture assays, to have anti-cancer properties [86]. Curcumin inhibits lipoxygenase activity and the production of cyclooxygenase 2. It prevents carcinogenesis from occurring by suppressing the cytochrome P-450 enzyme and increasing glutathione-S-transferase levels. It inhibits the promotion and progression of carcinogenesis while also influencing the formation of colon cancer cells with DNA mismatch repair defects [87]. As a result, curcumin may be a safe chemotherapeutic drug for tumors with DNA mismatch repair failure and microsatellite instability [88].

Lipid drug conjugates can help this approach in several ways. LDCs are lipids that have been conjugated to the parent medication either covalently or non-covalently. The drug’s lipophilicity and other characteristics are enhanced by conjugation with the lipid. This approach facilitates the distribution of medicine to the desired location. This section explains how to work with lipids produced from fatty acids. A drug molecule can enter the triglyceride (TG) deacylation–deacylation pathway when one of the fatty acids is replaced with a drug molecule. TG hydrolyzes into 2-monoglyceride (2-MG) and free fatty acid in the gastrointestinal lumen, where it is absorbed by erythrocytes and deacylated back to TG. Subsequently, the TG is converted into lipoprotein and stored in the lymphatic system [89]. These drug conjugates use lymphatic transport to improve medication absorption and target lymphatic drugs. Ex vivo testing can be used to investigate this by categorizing, locating, and isolating lymph nodes, as well as extracting the medicine to determine the drug concentration in the lymphatic system. The lymphatic system’s function in transporting dietary lipids from the intestine to the lymphatic capillaries allows the lipid drug mixture to efficiently use this pathway, integrate into the enterocytes, and arrive in the lymphatic capillaries, thereby overcoming the first-pass metabolism observed during oral drug administration [90].

**Figure 2:**
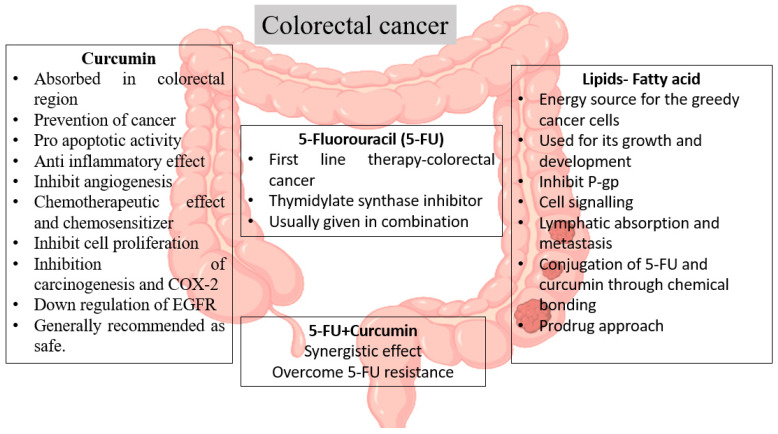
The mechanism of 5-FU lipid curcumin conjugates for the treatment of colon cancer.

This approach has the potential to treat metastatic malignancy because metastasis occurs primarily through the lymph nodes. Cancer cells use and metabolize more lipids than normal cells to proliferate rapidly. Fatty acids are primarily used as a basis of energy and as predecessors in a variety of biological processes. This lipophilic prodrug cleaves the lipids, leaving the parent drug behind, and is then delivered to tumor cells, causing cytotoxicity [91]. The mechanism of action of conjugates is depicted schematically in Figure 2. When it comes to delivering anticancer drugs to affected cells, nanomaterials have a high degree of selectivity and specificity. This combination has the potential to increase the cytotoxicity in colon cancer cells while leaving normal cells alone. This method can be accepted by the tumor microenvironment by improving retention and permeation (EPR). Flavonoids may have clinical applications through their inhibition of P-gp and, thus, their reversal of neoplastic MDR. Furthermore, they have the potential to produce synergistic effects. The combination of flavonoids, lipids, and chemotherapeutic drugs may be an effective treatment for colon cancer cells. Because of a lack of proper exercise and to the additional risks associated with the Western diet, the risk of colorectal cancer has increased in the modern era. As a result, this combination therapy involving lipids, flavonoids, and chemotherapeutic drugs, provides a foundation for reducing the main complications related to current therapies to some degree, in a cost-effective manner.

**Figure 3:**
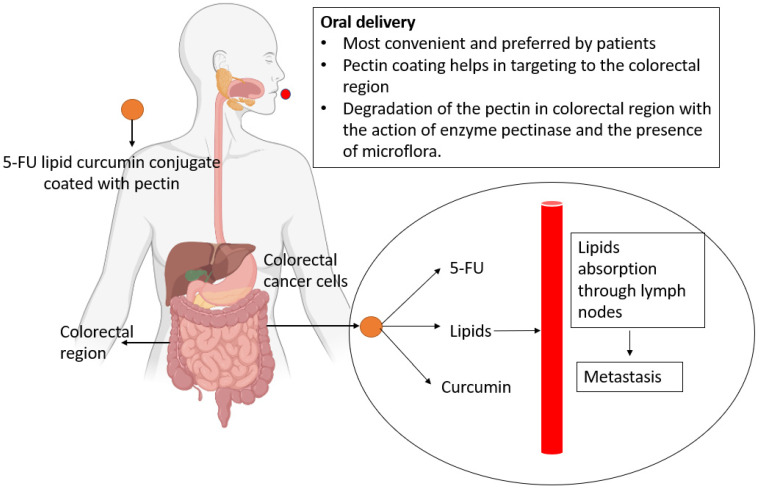
The delivery of drug conjugate through oral route and dissociation concept.

The ability of drugs to target the colon is a significant phenomenon in the treatment of colorectal cancer. In this case, pectin is used as a transporter for colon-specific drug delivery. The pectinase enzyme, which is produced by anaerobic bacteria in the colon, initiates the mechanism. Other pectin properties, such as film formation and pH sensitivity, make pectin-based drug delivery systems dependable and reproducible for colon-specific drug delivery [19]. To determine the fate of the lipid and allow the release of the drug conjugate, an in vitro lipolysis medium is developed. The medium’s calcium and bile aid in the digestion of lipids (lipolysis). This model can be used to study drug release due to bond dissociation [92]. To advance pharmacokinetic research and drug delivery, the mechanism of drug transport must be studied. The delivery of the drug conjugate through the oral route and the dissociation concept are given in Figure 3.

Because P-gp inhibits drug absorption, distribution, and elimination, the novel method’s invasion is required for effective drug delivery to the target area. P-gp is found on the biliary canalicular surfaces of hepatocytes, the luminal surfaces of jejunum and colon cells, the apical surfaces of the proximal tubular cells of the kidney, the endothelial cells of the blood–brain barrier, the apical membrane of the fetal membrane barrier function in the placenta, and other tissues, such as the adrenals, prostate, spleen, lungs, skin, heart, and skeletal muscles [93]. To study the efflux mechanism complex in drug resistance, MDR1 Madin Darby canine kidney (MDR1-MDCK), MDCK, and carcinoma cell lines can be used (Caco2). Comparisons of cell lines are drawn to achieve better outcomes.

Our theories address the following problems: (a) a novel approach to overcoming and changing MDR by inhibiting the P-gp efflux pump; (b) using conjugates in the lymphatic system to conquer the metastatic stage; and (c) improving treatment efficacy. If our theory is proven correct, it will deliver a safe, effective, and viable therapy that can be used even when colon cancer has spread to the metastatic stage. Because flavonoids have a synergistic effect on cancer cells [94], our anticipated formulation will reduce the drug’s dosing frequency even further [95]. This formulation may overcome the barriers to treatment failure and may offer a new approach to producing cytotoxic outcomes in cancer cells [96].

## 4. Conclusions

Cancer is a major cause of mortality globally. The major reasons for the failure of cancer treatment is the late diagnosis of cancer and multidrug resistance. Colorectal cancer is the world’s third most common cancer. The desire for fast food has increased the risk of colon cancer in the modern era. Because of the various side effects of treatment methods, such as alopecia, gastrointestinal tract irritation, and the possibility of secondary cancer (leukemia), a novel approach to reducing potential side effects became critical. The combination of flavonoids and chemotherapeutic agents offers a safe and effective therapeutic criterion that can address the issues associated with therapy failure while also producing a synergistic effect. We examined the resistance component of cancer in general and colorectal cancer in particular and postulated a concept that includes the possibility of combination therapy with 5-fluorouracil, curcumin, and lipids for the prospective management of colorectal cancer. In addition, we proposed a hypothetical combination of synthetic and natural chemotherapeutic drugs to treat colorectal cancer. In a hypothetical scenario, this may improve the treatment of colorectal cancer. The primary goal of this research was to develop a new combination of flavonoids and lipid drug conjugates for the treatment of colorectal cancer. This research hypothesis could be useful in the metastatic stage of cancer. If this theory is proven to be correct, we believe it could be used as a novel and cost-effective strategy in the treatment of colorectal cancer and, by changing the polymeric coating, it may even become a strategic approach to the treatment of other cancers.

## Data Availability

The data supporting the findings of this study are available within the article.
